# Miscellaneous Items

**Published:** 1886-09

**Authors:** 


### MISCELLANEOUS ITEMS.

**"Who** is that gentleman?"

"Dr. X.—a charming person, I assure you. If you only knew how he takes life—"

"Yes the life of others!"—*Tid-Bits.*

**"You are going** to erect a monument to your father's memory, I suppose?" "Oh, no! Monuments crumble and decay and are forgotten. We are going to do something that will keep his memory alive much louger." "What's that?" "We are going to fight in the courts for his property."

**A Drunkard's** nose is never an object of pride and joy, swollen, red, carbuncular, livid, loathsome. If the possessor of such a nose could conveniently examine his internal anatomy he would find that his brain, stomach, liver, lungs, heart and kidneys are exact counterparts of his nose. It might then seem to him a hint to quit drinking.

**"Good** morning, children," said a physician, as he met three or four little children on their way to school. "How do you do this morning?"

"We darn't tell you," replied the oldest of the crowd, a boy of eight.

"Dare not tell me," exclaimed the doctor, "and why not?"

"Cause papa says that it cost him fifty dollars last year to have you come in and ask how we were."

**The diet** of persons troubled with diarrhœs, or just recovering from such complaints, is often a cause of anxious inquiry. Here is a safe and simple dietary: Rice water or boiled milk, not too cold, as drink and nutrition; rice with boiled milk as food. The whites of eggs, well beaten up and sugared, plus spirit when needed, form an easy means of sustaining strength until recovery is established.

**A married woman** was telling a staid lady somewhat on the wrong side of fifty, of some domestic troubles, which she, in great part, attributed to the irregularities of her husband. "Well," said the old maid, "you have brought these troubles on yourself. I told you not to marry him. I was sure he would not make you a good husband." "He is not a good one, to be sure, Madam," replies the woman, "but he is a powerful sight better than none."

**Near Enough to Satisfy Her.**—It was a Guelph girl of whom the story is told that she refused to marry a most devoted lover until he should have amassed a fortune of $10,000. After some expostulation, he accepted the decree and went to work. About three months after this the avaricious young lady, meeting her lover; asked: "Well, Charlie, how are you getting along?" "Oh, very well indeed." Charlie returned, cheerfully, "I've got $18 saved." The young lady blushed and looked down at the toes of her walking boots, and stabbed the inoffensive earth with the point of her parasol. "I guess," said she faintly, "I guess, Charlie, that's about near enough."

**The Morning Bath.**—The cold or tepid sponge-bath, taken in the morning before breakfast, with friction to make the skin red, is one of the most health-giving actions we know. It promotes healthy circulation to the skin and all organs of our body, and keeps them in good condition and healthy in appearance. Some persons cannot indulge in such a "morning tub," by reason of peculiarity of constitution or from liver affections, and are unable to take the bath quite cold. It should, then, have just the chill taken off, but the skin should, in all instances, immediately after the bath, be thoroughly dried and rubbed with a coarse linen towel. No soap is used to this bath, and it is a good plan to have a warm bath about the temperature of 92° F. once a week. The brain is invigorated by the bath from the healthful stimulation of the nerves. If the person is not very strong, and the reaction is not perfect, a glass of very hot water taken after the bath, will prevent a chill.

**It is very** important for people to know which plants are poisonous and dangerous, especially now that so many are in the country on their vacation. One of our most popular groups of plants is the butter-cup, but many of these are sufficiently acrid to produce blisters when applied to the skin. The poisonous sumac is probably the most dangerous of all our native plants which poison through contact. Avoid all sumac which has not red berries, and you will be safe from poison. The common horse-chestnut is used in Europe as food for horses and cattle, and yet the small horse-chestnut of the south, "Buckeye," is claimed to be poisonous. The laburnum or gold chain, a common garden plant, has roots which taste like licorice and which thus tempt children to chew it. It is, however, a dangerous poison to eat. Water hemlock, fox-glove or digitalis, aconite, Jamestown weed or stramonium, common poke weed, skunk cabbage, poison ivy, and various other common weeds or shrubs, including some mushroons, are rankly poisonous and should be avoided.

**The Girl of To-day.**—The girl of to-day is a busy, useful worker. She is generally proficient in needle-work. She can not only alter her own dresses, but cut and make them and her underclothing as well.

She has a knack of trimming her hats and furnishing her wardrobe, and does her full share at helping the dressmaker who comes to assume charge of the spring and fall sewing. She understands the various branches of mending, and takes that division of labor off her mother's hands, as well as the care of parlors and dining-rooms, the arrangement of flowers, the supervision of manners and apparel of the younger children, and sometimes their studies, too. Let full justice be done to the "girl of the period," or rather let there be a clear comprehension of what should be really represented by that much-abused phrase. It is not fair to take the weakest specimen of the sex as types of a class comprising earnest workers, with strong conceptions of life, its responsibilities and burdens, and a steady purpose to bear them according to the best of their ability.—*Toronto Truth.*

---

**The lack** of a pronoun of common gender in our language occasionally causes the formation of some amusing sentences. Thus, a writer, speaking of the natives of Point Barrow, observes: "If any thing was given a child it showed its appreciation thereat sometimes in words, but more often in smiles and by informing its play-fellows that he or she had been shown especial favors by the great white captain." An educational article, referring to school-children of either sex, says: "We believe there was not a single individual who did not understand the full purpose of their act." The pronoun *whoso* might be used more frequently than it now is in cases of this sort. The following is a good sentence in point: "The English language itself, with its treasures of great books, is, in my opinion, quite capable of furnishing to whoso will study it rightly all the indispensable means of mental culture."—*The Student.*

There is no writer in our language who has not continually felt the want of a pronoun significant of either gender. The extreme awkwardneas of continuing the form of expression "he or she" has been too long allowed. Surely a people who are always apt at inventing a new word, to supply a new and unexpected demand, should not be put to its trumps in a matter so simple as the foregoing would appear to be. Why not adopt one by common consent, regardless of any philological connection or derivation. Almost anything will do. When our first parent named all living objects in their turn, quite off-handed, he did not run around to borrow a dictionary to see if this or that would be just the thing. He went at it in a business way and never once made a mistake. Suppose we say het or hom or hes. One will do as well as another after we get used to it.

